# PPARs: Interference with Warburg' Effect and Clinical Anticancer Trials

**DOI:** 10.1155/2012/304760

**Published:** 2012-05-08

**Authors:** Joseph Vamecq, Jean-Marie Colet, Jean Jacques Vanden Eynde, Gilbert Briand, Nicole Porchet, Stéphane Rocchi

**Affiliations:** ^1^Inserm, HMNO, CBP, CHRU Lille, 59037 Lille, France; ^2^Biochemistry and Molecular Biology, HMNO, CBP, CHRU Lille, 59037 Lille, France; ^3^Department of Human Biology and Toxicology, Faculty of Medicine and Pharmacy, UMons, 7000 Mons, Belgium; ^4^Organic Chemistry Laboratory, Faculty of Sciences, UMons, 7000 Mons, Belgium; ^5^Inserm U1065, IFR 50, Mediterranean Center of Molecular Medicine, 06204 Nice, France

## Abstract

The metabolic/cell signaling basis of Warburg's effect (“aerobic glycolysis”) and the general metabolic phenotype adopted by cancer cells are first reviewed. Several bypasses are adopted to provide a panoramic integrated view of tumoral metabolism, by attributing a central signaling role to hypoxia-induced factor (HIF-1) in the expression of aerobic glycolysis. The cancer metabolic phenotype also results from alterations of other routes involving *ras, myc, p53,* and *Akt* signaling and the propensity of cancer cells to develop signaling aberrances (notably aberrant surface receptor expression) which, when present, offer unique opportunities for therapeutic interventions. The rationale for various emerging strategies for cancer treatment is presented along with mechanisms by which PPAR ligands might interfere directly with tumoral metabolism and promote anticancer activity. Clinical trials using PPAR ligands are reviewed and followed by concluding remarks and perspectives for future studies. A therapeutic need to associate PPAR ligands with other anticancer agents is perhaps an important lesson to be learned from the results of the clinical trials conducted to date.

## 1. Introduction

 Nowadays, cancer therapy offers strategies that do not primarily target nuclear DNA integrity, repair, duplication, or synthesis. These approaches address an event that is specific to cancer cells (inhibition/neutralization of overexpressed tyrosine kinase, for instance) or disrupt universal features of cancer development such as neovascularization. Though the therapeutic target should ideally be essential in cancer cells but not in normal cells, treatment may in turn restore sensitivity or remove resistance to physiological processes such as the apoptotic pathways. Various mechanisms underlying the anticancer actions of PPAR effects and ligands have previously been developed in other issues of this journal [1–7], as well as some controversial activity, notably regarding PPAR*β*/*δ*-driven effects [8–10].

Besides neovascularization, other characteristics common to many cancers are currently targeted by “mitocans” (drugs destabilizing tumoral mitochondria to induce cell death by cytotoxicity or apoptosis) [11, 12] and what might be called “metabocans” (drugs disrupting tumoral metabolism), acronyms of “mitochondria and cancer” and “metabolism and cancer”, respectively. The compounds covered by these acronyms may, however, overlap.

The desired endpoint of any anticancer therapy is a combination of optimal efficacy, minimal side effects, and prevention of resurgence. In practice, it is sometimes far from being met, some cancers being still incurable or hardly resolvable. The optimization of this goal resides in targeting features distinguishing more categorically cancer cells from normal cells. Encouraging examples have been provided by tyrosine kinase-directed antibodies or inhibitors which despite chemoresistance have served as templates to boost the development of novel anticancer drugs that target, for instance, breast cancers overexpressing HER-2 surface receptor or chronic myeloid leukemia overexpressing BCR-ABL tyrosine kinase, a fusion gene product [13]. The anticancer activity of drugs antagonizing neovascularization by interfering for instance with vascular endothelial growth factor (VEGF) signaling represents another development in cancer-targeted therapy [14]. Mitocans also currently represents a promising approach [11]. Disrupting cancer cell metabolism to induce cell death (*via* apoptosis, necrosis, or both) represents another elegant approach. “Metabolic therapy of cancer,” a concept aimed at controlling malignant behavior, was discussed before apoptosis came onto the scene [15, 16]. It would now be better to speak of metabolism disruption-driven cell death. Several drugs could be referred to as mitocans, metabocans, or aberrocans (disruption of biased signaling), for instance, monoclonal antibodies or kinase inhibitor-based drugs, and many other such drugs are being developed at present [17]. A major difficulty is targeting cancer cell signaling aberrance(s) without affecting kinase functions that are of crucial importance for normal cells.

**Figure 1:**
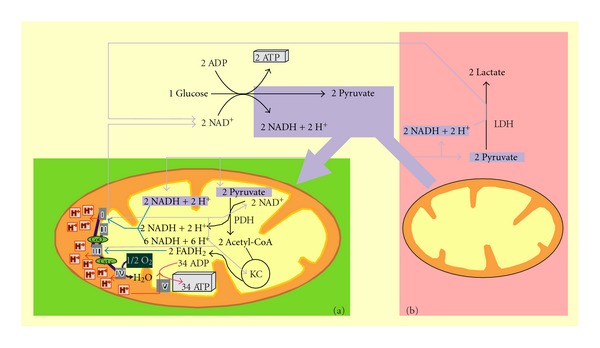
Metabolism of glycolysis-derived NADH and pyruvate in normoxia (a), anoxia and cancer (b). (a) Normoxic normal cells classically oxidize glucose to completion. Cytosolic enzymes convert 1 molecule of glucose to 2 molecules of pyuvate and along with 2 ATP and 2 NADH. Mitochondrial oxidations of glucose-derived pyruvate and NADH involve pyruvate dehydrogenase (PDH), Krebs cycle (KC), and respiratory chain electron transfer/oxidative phosphorylation (OXPHOS) complexes I, II, II, IV, and V, yielding classically 34 ATP. Complete oxidation of glucose therefore results in the production of 36 (2 cytosolic + 34 mitochondrial) ATP. (b) The contribution of mitochondria to glucose oxidation is disrupted in anoxic normal or cancer cells by the arrest of mitochondrial respiration (lack of oxygen in anoxia) and in normoxic and anoxic cancer cells by different convergent mechanisms. Among these, reduced pyruvate dehydrogenase activity may result from overexpressed pyruvate dehydrogenase kinase 1 and limited access of pyruvate to the mitochondria due to the closed state of mitochondrial outer membrane voltage-dependent anionic channel (VDAC). Reduced activities of the respiratory chain complexes I and IV and muted Krebs cycle enzymes may be also encountered. Pyruvate, formed intracytosolically from glucose *via *glycolysis, is no longer oxidized in mitochondria and is metabolized by cytosolic lactate dehydrogenase. In cancer cells, it must be stressed that the metabolic events mentioned above take place in the context of fuel producing glycolysis in which cytosolic net ATP formation occurs. In turn, the glycolytic flux may be blocked at the pyruvate kinase step (see Figure 4), resulting in biosynthetic precursor-producing glycolysis with little or no net glycolytic ATP or pyruvate production. Cytosolic pyruvate is then provided *via* other routes (precursors other than glucose) including serinolysis and glutaminolysis, pathways which refer to conversions of serine and glutamine to lactate, respectively.

Cancer cells express a metabolic phenotype that is distinct from normal cells as emphasized by Figure 1 which illustrates the contributions of glucose oxidation to ATP synthesis in normal cells under normoxia and in hypoxic/anoxic or cancer cells (cancer cells will be considered as having lazy mitochondria throughout this review) [18, 19]. In contrast to the normal aerobic glucose metabolism pathway which uses mitochondrial oxidation, cancer cells develop Warburg's effect [20, 21], in which aerobic glycolysis is very much increased and for which drug-driven disruption might lead to minimal side effects. Because Warburg's effect involves most if not all cancers, its disruption in a way and extent that cannot be counterbalanced by cancer cells might then resolve the malignant process, independently of its origin.

The ubiquity of Warburg's effect in tumors has been evidenced by positron emission tomography scan imagery of ^18^F-deoxyglucose (FDG-PET), a glucose analogue transported and phosphorylated in cells without further metabolism for several decades. The tight link existing between tumoral status and FDG-PET data might confirm the pertinence of any therapeutic strategy aimed at disrupting tumoral metabolism. Interestingly, 2-deoxy-D-glucose and analogues are currently being developed as a drug template for treating cancer by competing with the metabolic feature that it was first used to demonstrate when used in its labeled form (^18^F-deoxyglucose) in FDG-PET. More precisely, 2-deoxy-D-glucose presents anticancer properties and may potentiate the efficacy of prototype anticancer drugs [22].

Targeting tumoral metabolism in a way that cannot be counterbalanced by cancer cells is not, however, an easy task. Pragmatically, this strategy requires a general integrated view of tumoral metabolism because it is not a single metabolic step that is altered but the entire energetic metabolism that works on a pattern profoundly affected in cancer (versus normal) cells. This metabolic *modus vivendi* results from permissive alterations in cell signaling among which HIF-1 routes. Although it would be an oversimplification to consider that tumoral metabolism is close to anaerobic metabolism, it may help in understanding how, step by step, it is organized in comparison with normal metabolism.

An appraisal of this organization in relation to Warburg's effect is therefore provided in the following two chapters by explaining step by step the metabolic and signaling articulations that exist between tumoral glycolysis and cancer mitochondria, and then the particular role of tumoral pyruvate kinase. The properties of PPARs in relation with Warburg's effect and clinical trials using PPARs as anticancer agents, all mechanisms comprised, will be then reviewed.

## 2. Tumoral Glycolysis and Mitochondria

### 2.1. General Characteristics

Different patterns of glucose metabolism are observed in normal cells in normoxia and hypoxia/anoxia. Under oxygen, one glucose molecule is oxidized in the cytosol into two pyruvates which enter mitochondria for decarboxylation by pyruvate dehydrogenase forming acetyl-CoA which is further oxidized *via* the Krebs cycle (Figure 1). This aerobic oxidation of glucose yields approximately 36 molecules of ATP. Hypoxic/anoxic normal cells develop only the glycolytic contribution to glucose oxidation, converting one glucose into two pyruvates that are then reduced locally into lactates by lactate dehydrogenase. This reaction recycles the NAD^+^ required for glycolysis to proceed. The net result is here two (instead of 36) molecules of ATP formed by oxidation of one molecule of glucose (Figure 1).

Normoxic oxidation of glucose in cancer cells resembles that observed in hypoxic/anoxic normal cells. This similarity is emphasized when they are referring to as aerobic (Warburg's effect) and anaerobic glycolysis, respectively. The tumoral metabolic phenotype results from alterations in several regulatory pathways including p53, myc, ras, Akt, and HIF-1 signaling pathways [23–26]. The biased nature of signaling pathways, especially the HIF-1 signaling pathway, the role of pyruvate kinase and the fact that cancer cells have to cope with hypoxia influence tumoral metabolism and provide a general outline for the process. The ability of cancer cells to function like anaerobic cells despite normoxia might explain their extraordinary tolerance to anoxia.

### 2.2. Cell Signalling Involving HIF-1

**Figure 2:**
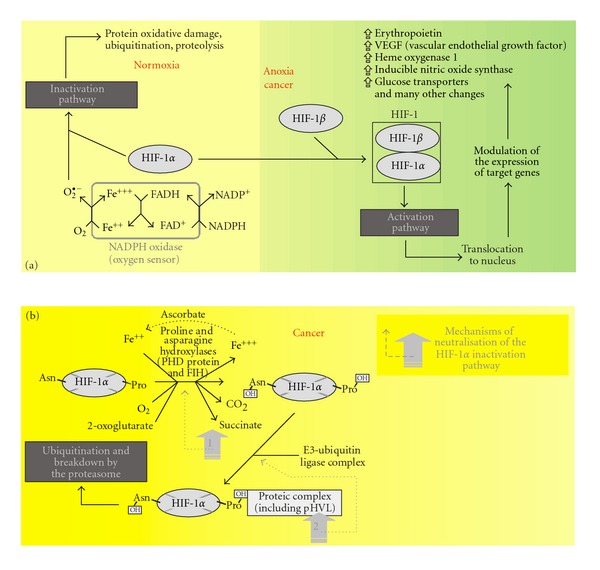
Activation and inactivation pathways for hypoxia-induced factor 1 (HIF-1) in normoxia, hypoxia/anoxia, and cancer. (a) In normoxic normal cells, molecular oxygen (O_2_) sensing by membrane NADPH oxidase results in formation of superoxide radical anions and subsequently in the control of the HIF-1 signaling pathway *via* proteolytic degradation of the HIF-1*α* subunit. These events prevent the recruitment of functional HIF-1 which results from heterodimerization of HIF-1*α* and *β* subunits. In anoxia/hypoxia, a severe drop in the levels of NADPH oxidase-driven superoxide radical anion prevents oxidative and proteolytic damage of the HIF-1*α* subunit which then becomes available to form functional HIF-1 and results in signaling activation. In cancer cells, the HIF-1 signaling pathway may be overexpressed *via,* for instance, mechanisms illustrated in Panel b. (b) This panel details hydroxylation and the subsequent steps of the HIF-1 inactivation pathway and its disruption in cancer cells. Mechanisms that neutralize the HIF-1 pathway in cancer cells may include alteration of gene expression for succinate dehydrogenase and fumarase (the net results of which are a rise in succinate levels which interfere with the hydroxylation steps by product inhibition) (mechanism 1) and for von Hippel-Lindau protein (mechanism 2) (preventing formation of E3-ubiquitin ligase proteic complex and hence HIF-1*α* inactivation). Other comments are in the text.

The anaerobic-like metabolic phenotype observed in normoxic cancer cells may result from biased sensing of oxygen by the HIF-1 signaling pathway. HIF-1 inactivation and activation pathways [27–29] are illustrated in Figure 2. In normal cells, oxygen blocks the activation of HIF-1 signaling. Exposure of cells to oxygen downregulates functional HIF-1 by restricting the availability of its *α* subunit. More precisely, molecular oxygen is sensed by cell membrane NADPH oxidase which reduces it to superoxide. This species oxidatively damages the HIF-1*α* subunit, initiating degradation by the proteasome (Figure 2). Functional HIF-1, following heterodimerization of the *α* and *β* HIF-1 subunits in the cytosol, undergoes nuclear translocation and activates target genes. Molecular oxygen normally “paralyzes” HIF-1 signaling by inducing depletion of its *α* subunit. In hypoxic/anoxic normal cells, the *α* subunit is little oxidized/degraded, favoring functional HIF-1, nuclear translocation, and gene activation. These events give rise to the glycolytic phenotype in which, in contrast to oxidative phenotype, mitochondrial oxidations do not contribute to glucose oxidation. Normal cells develop oxidative and glycolytic phenotypes in normoxic and anoxic conditions, respectively.

In normoxic cancer cells, HIF-1 inactivation is disrupted, and hence HIF-1 signaling is enhanced, giving a rise to a glycolytic phenotype despite the presence of oxygen. Figure 2(b) details hydroxylation and the subsequent steps of the inactivation pathway. In normal normoxic cells, oxygen contributes to HIF-1 inactivation by initiating superoxide-driven oxidative damage to the HIF-1*α* subunit (see Figure 2(a)) and by promoting hydroxylation steps (also considered as oxygen sensors). In cancer cells, formation of the complex involving von Hippel Lindau protein (mutations affecting the E3 ubiquitin complex ligase gene) may be deficient [30]. Mutations may also affect the succinate dehydrogenase and/or fumarase genes [31, 32], and the resulting succinate accumulation alters hydroxylation steps by product inhibition. These genes (von Hippel Lindau protein, succinate dehydrogenase, and fumarase genes) represent tumor suppressor genes, inborn errors of which favor cancer development [31, 32].

### 2.3. Metabolism Compartmentalization Induced by HIF-1 Signaling

**Figure 3:**
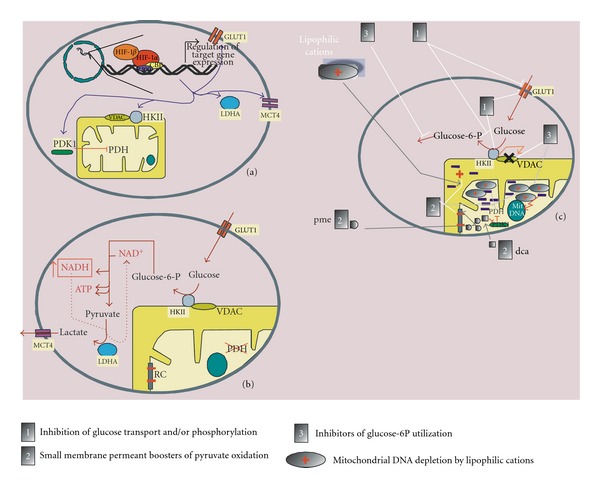
Illustration of how biased HIF-1 signaling in cancer cells may cause Warburg' effect and lead to modified protein content and subcellular localization (a), metabolism (b), and cancer-specific therapeutic opportunities (c). The role of aberrant HIF-1 signalling in cancer cells by encoding proteins (a) which modify the intermediary metabolism in a way that favors the emergence of glycolytic metabolism even in normoxic conditions (b). Note the convergence of the HIF-1-driven increase of proteins, convergence which favors tumor vascularization (Figure 2(a)), and aerobic glycolysis (this figure). Panel (c) illustrates therapeutic opportunities related to cancer cell metabolism. Inhibition of glucose transport and activation disrupts Warburg's effect, depriving cancer cells of their preferential metabolic substrate. Inhibition of hexokinase may in addition lead to its detachment from mitochondrial VDAC. Blocking the utilization of glucose 6-phosphate by increasing its concentrations secondarily leads to hexokinase inhibition and hence its detachment from VDAC (reopening this channel). Direct interaction with VDAC might also disrupt the closed state of the channel. The closed channel may be, however, bypassed by small permeant compounds such as pyruvate methyl ester (pme) and dichloroacetate (dca). When VDAC is closed, pyruvate methyl ester, in contrast to pyruvate, can enter the mitochondrial matrix where an esterase produces pyruvate, following this the action of residual pyruvate dehydrogenase generates an electron flux towards the respiratory chain (at the level of complex I), a feature capable of triggering mitochondrial apoptosis. Dichloroacetate inhibits pyruvate dehydrogenase kinase activity and then restores substantial pyruvate dehydrogenase activity and subsequent flux towards respiratory chain. Lipopphilic cationic compounds are attracted by cancer cell mitochondria which present abnormally high negative electric charges consequently to reduced electron chain proton efflux and to the closed state of VDAC (resulting in accumulation of small negative metabolites trapped within the mitochondria). Lipohilic cations may cross mitochondrial membranes, bypassing VDAC and being insensitive to the closed state of this channel. The intramitochondrial accumulation of lipophilic cations induces destruction and depletion of mitochondrial DNA. Abbreviations are GLUT1, glucose transporter 1; LDHA, lactate dehydrogenase A; MCT4, monocarboxylate transporter 4; PDH, pyruvate dehydrogenase; PDK, pyruvate dehydrogenase kinase1; HKII, hexokinase II; VDAC, voltage-dependent anionic channel; pme, pyruvic methyl ester; dca, dichloracetate; RC, respiratory chain.

Permanent activation of the HIF-1 pathway and some other signaling pathways in cancer cells enhance the expression of genes encoding proteins involved not only in tumoral angiogenesis and substrate supply (for instance, erythropoietin and VEGF) (see Figure 2(a)) but also in the glycolytic phenotype (Figure 3). Tumoral glucose metabolism increases cytosolic NADH which is oxidized back to NAD^+^ by cytosolic lactate dehydrogenase which converts pyruvate into lactate (Figure 3). Figure 3(b) illustrates how tumoral glycolysis starts at the mitochondrial outer membrane where hexokinase type II (HKII) interacts with the voltage-dependent anionic channel (VDAC), also located in this membrane, *via* a binding domain [33]. 

### 2.4. Metabolic Impact of Tumoral Glycolysis

The oxidation of glucose to pyruvate (glycolysis) yields less energy (only 2 ATPs instead of 36 ATPs, see above) than glucose oxidation to CO_2_ (glycolysis plus mitochondrial oxidations). Glycolytic mobilization of ATP as developed by cancer cells is, however, faster than in normal cells. Therefore, despite a lower recovery, energy mobilization is faster in tumoral cells as attested by the increased uptake of ^18^F-deoxyglucose observed in FDG-PET scans of patients with malignant tumors.

Normal cell mitochondria are positively charged by the respiratory chain-driven proton gradient. In contrast, cancer cell (lazy) mitochondria are negatively charged on account of the accumulation of negative molecules due to the closed state of VDAC.

In normal mitochondria, VDAC is open, allowing the exchange of small solutes (pyruvate and other negatively charged compounds with a molecular weight inferior to 1.5 KDa) [34]. In cancer cell mitochondria, VDAC is closed by a rise in cytoplasmic NADH and its interaction with HKII [34], therefore, inhibiting the exchange of small solutes. The VDAC-HKII complex can dissociate and VDAC may reopen subsequently following an increase in the levels of glucose-6-phosphate which is produced by glucokinase or hexokinases. Figure 3(c) presents various therapeutic opportunities based on these differences between cancer and normal cells.

## 3. Tumoral Pyruvate Kinase

The ultimate goal of sustained glycolytic rates in cancer cells is not only to provide an advantageous mode of energy mobilization that still works when the cell needs to move or when oxygen in the environment is low. It is also to ensure anaplerosis of nucleic acid synthesis and other biosynthetic pathways by supplying ribose phosphate moieties. This supply is provided by articulations existing between glycolysis and the pentose phosphate pathway, with the development of a nonoxidative pentose phosphate cycle. Ribose phosphate supply and lactate formation from glucose are distinct exclusive endpoints. When glycolytic intermediates enter the pentose phosphate route (to form ribose 1-P and hence ribose 6-P) or other biosynthetic pathways branching off from glycolysis, their carbon skeleton can no longer be used for pyruvate and lactate formation. Reciprocally, when glucose is converted to pyruvate (2 pyruvates per glucose), it can no longer be used for ribose phosphate synthesis and other biosynthetic processes. Because proliferative cells need energy, cell death would occur if no regulatory mechanisms were implemented when nutrient supply is limited.

Several sensors enable cells to adapt cellular growth and proliferation to nutrient supply. Pyruvate kinase, which is responsible for the net ATP formation during glycolysis, represents such a key sensor. It is, for instance, inhibited by ATP (high when nutrient supply is high) and activated by AMP and Pi (high when nutrient supply is low), directing differently phosphoenolpyruvate (and hence glucose) towards biosynthetic pathways and ATP formation, respectively. Cells have developed an elegant means to avoid pyruvate kinase inhibition when the glucose supply is low and on the opposite pyruvate kinase activation when the glucose supply is high. This regulator, which couples the glucose cell supply to pyruvate kinase activity, is fructose 1,6-biP, a potent physiological activator of pyruvate kinase [35]. Fructose 1,6-biP is itself dependent on fructose 2,6-biP, the powerful stimulator of phosphofructokinase [36].

Pyruvate kinase exists in various isoforms, L in the liver and kidneys (gluconeogenic metabolism), R in red blood globules; M1 in muscles and the brain (tissues in which rapid mobilization of energy may be required), and M2 in the lungs and tissues with nucleogenic metabolism (increased nucleic acid synthesis) including normal proliferating cells, stem cells, and tumoral cells [35]. Normal cell and tumoral M2 isoforms differ in their quaternary structures which are tetrameric and prominently dimeric, respectively [35]. Tetrameric (normal) and dimeric (tumoral) forms of M2 pyruvate kinase exhibit distinct kinetic properties and induce distinct processes. Tetrameric M2 pyruvate kinase favors the conversion of phosphoenolpyruvate to pyruvate and net glycolytic ATP production, driving glycolytic phosphometabolites towards fuel production. In contrast, dimeric (tumoral) M2 pyruvate kinase is inactive in the presence of physiological concentrations of phosphoenolpyruvate and does not promote net ATP formation so glycolytic phosphometabolites are channeled towards biosynthetic processes including nucleic acid, aminoacid, sialic acid, and phospholipid biosynthesis.

**Figure 4:**
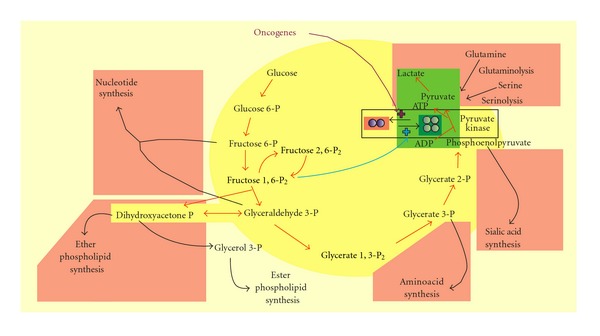
Tetrameric M2 pyruvate kinase-driven fuel-generating (green panel) and dimeric M2 pyruvate kinase-driven biosynthetic precursor-generating (red panels) glycolysis (yellow panel) in cancer cells. Tumoral M2 pyruvate kinase exists in a dimeric inactive form that blocks pyruvate and ATP formation from glucose. It induces the accumulation of energy-rich phosphometabolites found upstream in the glycolytic pathway and off which biosynthetic processes may branch. Interconversion to the active tetrameric form of the enzyme may occur when the glucose supply is high, leading to a rise in fructose 1,6 bisphosphate which stimulates this tetrameric conversion. When glucose levels are high, cancer cells may then produce energy at the same time as supplying biosynthetic pathways. In this situation, the rise in glycolytic intermediary rich energy phosphometabolites results not from a block located downstream of their production but from an increased load of glycolysis by glucose. When glucose levels fall again, the subsequent decrease in fructose 1,6 bisphosphate results in recruitment of the dimeric inactive form of M2 pyruvate kinase. In this case, both energy and pyruvate production (and hence formation of lactate) by the tumor may derive from the catabolism of aminoacids such as glutamine and serine. The latter sets of metabolic reactions, by analogy with glycolysis for glucose to lactate production, are referred to as glutaminolysis and serinolysis, respectively. The supply of these aminoacids to the tumor is associated at distance with notably muscle proteolysis, explaining the progression of patients towards a cachectic state when the tumor gains in growth and development. Cachexy may be also favored by energy wasting associated to uncoupling of mitochondria in some cancer cell lines. Except with tumors such as insulinoma and hepatoma, for instance, no hypoglycaemia is, however, induced in patients since a sustained production of lactate is ensured by the tumor from these aminioacids. Lactate may be recycled to glucose by gluconeoformator cells, mainly hepatocytes (Cori's cycle). Biosynthetic pathways branching off glycolysis include sialic acid, nucleic acid, aminoacid, ether glycerolipid, and ester glycerolipid anabolic pathways. The latter pathway is not emphasized here by inclusion in a red panel because in many cancer cell lines glycerol phosphate dehydrogenase is deficient thus reducing the availability of glycerol 3-phosphate and limiting incorporation of neoformed fatty acids into lipids. Fatty acid synthase is often overexpressed, and, along with the removal of fatty acids (known for immunosuppressive properties) outside the cell, it allows tumoral cells to cope with the massive rise in glycolysis-driven NADH and proton (H^+^) formation (glyceraldehyde 3-phosphate dehydrogenase step), avoiding excess acidification and consequent cell death. The threshold for reversible interconversion of tetrameric to dimeric M2 pyruvate kinase may be lowered by oncogenes in favor of the dimeric form. As mentioned in the text, the tetrameric active form is part of the glycolytic complex (a complex which groups most glycolytic enzymes for optimal metabolic function and energy production) whereas the dimeric form separates from this glycolytic complex.

Therefore, in tumoral cells exhibiting high glucose consumption rates, the inactive dimeric form of M2 pyruvate kinase allows glycolytic phosphometabolites to accumulate and subsequently be used for biosynthesis. Ribose 5-P synthesis branches off from glycolysis *via* the transketolase/transaldolase reaction (nonoxidative pentose phosphate cycle) because the oxidative pentose phosphate cycle is inhibited by high levels of fructose 1,6 biP (consecutive to increased glucose supply and reduced pyruvate kinase activity) [35]. The role of M2 pyruvate kinase in cancer has been reviewed in detail elsewhere [35]. In cancer cells, the shift from dimeric to tetrameric M2 pyruvate kinase is dependent on fructose 1,6 BiP levels; according to a threshold-driven regulation mechanism the enzyme is converted from the dimeric to the tetrameric form when concentrations exceed the threshold and inversely when concentrations are below the threshold. The threshold itself is subject to modulation by signaling molecules such as oncogene products which may lower the threshold [35]. The contribution of the dimeric and tetrameric forms of tumoral M2 pyruvate kinase to Warburg's effect is illustrated in Figure 4. 

## 4. PPARs, Metabolism, and Inflammation

**Figure 5:**
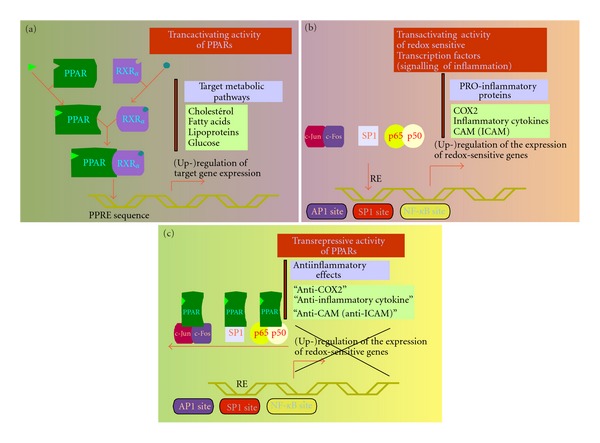
Modulation of gene expression by PPARs and inflammatory transcription factors: transactivation and transrepression mechanisms. The activation of PPARs (a) and inflammatory transcription factors (b) modulates gene expression *via* specific response elements (RE), resulting mainly in the upregulation of proteins involved in several metabolic and inflammatory pathways, respectively. The two routes may connect through protein-protein interaction upstream to DNA-protein interaction (c). This transrepressive action of a pathway on the other is a basis for the anti-inflammatory/antioxidative properties of PPARs, and though less often mentioned, for the anti-PPAR properties of inflammatory/oxidative processes. Abbreviations: COX2, cyclooxygenase 2; ICAM, inducible cell adhesion molecule; PPRE, PPAR-responsive element; RXR*α*, retinoid acid X receptor *α*. The mechanisms by which metabolic changes and anti-inflammatory properties induced by PPARs interfere with the Warburg's effect and cancer development are considered in the text.

PPARs are nuclear receptors which influence many aspects of cell physiology. Their action is pleiotropic and impacts in diverse ways the signalome, the metabolome, and the cell cycle. The three PPAR isoforms have metabolic effects and anti-inflammatory properties. Metabolic effects of PPARs result notably from the transactivation properties of ligands involving the PPAR-RXR*α* heterodimer, whereas the anti-inflammatory effects of ligands result mainly from the ability of activated PPARs to combine with transcription factors involved in inflammation signaling [37].  Figure 5 illustrates this dual ability of PPARs to control the activation of metabolic proteins by heterodimerizing with RXR*α* and hence binding to DNA, and to downregulate inflammatory pathways by interacting with so-called redox transcription factors without further binding to DNA of the PPAR/inflammatory transcription factor complex. These two types (metabolic and anti-inflammatory) of effects induced by PPARs could contribute to Warburg's effect. Nevertheless, although cumulative evidence supports the ability of metabolic events such as those induced by PPARs to interfere with Warburg's effect, the impact of anti-inflammatory events similar to those achievable by PPARs and their ligands on Warburg's effect has been pointed to recently and is still little documented.

## 5. PPARs and Metabolism of Inflammatory Fatty Acid Derivatives

**Figure 6:**
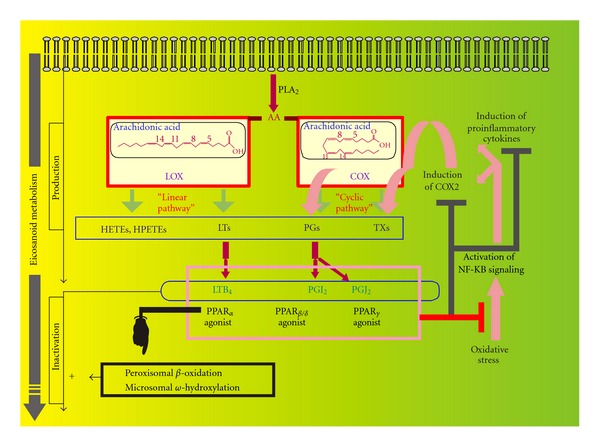
Arachidonic acid metabolism as a provider of physiological PPAR ligands. The figure illustrates in a nonexhaustive way that arachidonic acid (AA) may generate various lipoxygenase (LOX) (hydroxyeicosatrienes, HETEs; hydroperoxyeicosatrienes; HPETEs, leucotrienes, LTs) and cyclooxygenase- (COX) (prostaglandins, PGs; thromboxanes, TXs) derived metabolites. Among these metabolites, LTB4, prostacyclin (PGI2), and 15-deoxy PGJ2 (PGJ2) represent ligands of each of the three PPAR isoforms, PPAR*α*, *β*, and *γ*, respectively. Subsequent physiological activation of PPARs impacts the formation of such ligands *via* a negative feedback loop by increasing their degradation and lowering their formation. Indeed, PPAR*α* activation stimulates microsomal *ω*-hydroxylase (in humans) and peroxisomal *β*-oxidation (in rodents, for instance but not in humans) and hence degradation of each of the precited PPAR ligands. Each of the PPARs may upregulate the activity of antioxidant enzymes and downregulate inflammatory proteins. Preventing induction of cyclooxygenase type 2 is a way to counteract inflammation-driven increase in arachidonic metabolites which are cyclooxygenase-dependent ligands of PPARs. The interest of a PPAR-based therapy in preventing the synergism between stromal cell inflammation and tumor development/invasion is explained in the text.

The link between PPARs and eicosanoid metabolism is strong, tight, and reciprocal. Indeed, whereas eicosanoid metabolism may form physiological PPAR ligands [38], PPARs themselves regulate eicosanoid metabolism. As illustrated in Figure 6, at least three physiological PPAR ligands are issued from arachidonate metabolism, namely LTB4, PGI2 and 15-deoxy-PGJ2 which activate PPAR*α*, *β*/*δ*, and *γ*, respectively. By their transrepressive activity, the activation of PPARs prevents induction of COX2 *via* a physiological feedback loop. This loop limits the additional production of arachidonate-derived inflammatory mediators in response to oxidative stress (generated for instance by the recruitment of inflammatory cells) by controlling the level of induction of COX2 [37]. As COX2 activity increases, more physiological ligands of PPAR*β*/*δ* and PPAR*γ* are produced which potentiate the transrepressive activity of these isoforms on inflammatory signaling-driven stimulation of COX2 gene expression (in practice, agonists of each of the three main PPAR isoforms may induce this antiinflammatory effect).

Physiological activation of PPAR*α* by the LOX-derived eicosanoid metabolite LTB4 also takes place in the context of a negative metabolic feedback loop when taking into account the ability of this PPAR isoform to induce leukotriene-inactivating pathways. These inactivating pathways may also concern COX-derived metabolites and include peroxisomal *β*-oxidation and fatty acid *ω*-hydroxylation. Prostaglandins and leucotrienes may be inactivated by carbon chain shortening corresponding to one or two peroxisomal *β*-oxidation cycles [39]. They may also be inactivated by hydroxylation at the terminal carbon position of their fatty acid chain by a fatty acid *ω*-hydroxylase (using NADPH+H^+^, O_2_, and cytochrome P450 of the CYP4A or CYP4F subfamilies) [40, 41]. In rodents, the two pathways are activated by PPAR*α* whilst in humans peroxisomal *β*-oxidation does not seem to be induced [39]. In contrast, fatty acid *ω*-hydroxylase activity and CYP4A levels are enhanced by PPAR*α* activation in the humans [42].

## 6. PPARs, Inflammation, Angiogenesis, and Warburg's Effect

The modulation of inflammatory events by PPARs in relation to Warburg's effect is starting to be described. Though the role of inflammation in cancer has been largely documented, the relations between inflammation, and Warburg's effect are still currently the topic of a limited number of scientific papers. Common pathogenesis events involving the production of oxidant species would lead to the concurrence of cancer cells with the Warburg's effect phenotype and of an inflammatory microenvironment of the tumor which both favor cancer progression. In a recent paper, Pavlides et al. [43] elegantly illustrated this situation by showing that the loss of stromal caveolin-1 leads to oxidative stress and hypoxia, inducing HIF which favors Warburg's effect and triggering NF-*κ*B signaling which, *via* the activation of inflammatory pathways, induces inflammation. These cell signaling routes cause nitric oxide overproduction, mitochondrial dysfunction and ischemia mimic. Pavlides et al. [43] demonstrated that, in fact, mice lacking stromal caveolin-1 represented an animal model displaying the tumor stroma without the tumor, in other words the stromal ground which pathophysiologically dialogs and interacts with Warburg phenotyped cancer cells during *in situ* cancer growth and progression. These results might indicate that stroma cell inflammation and Warburg phenotyped cancer cells coexist in a kind of symbiosis in which each cell type takes advantage of being in the presence of the other. Because of their anti-inflammatory properties, PPARs should, therefore, affect this symbiosis between cancer cells and their stromal microenvironment and hence might impair tumoral growth and cancer progression. It is possible that part of the anticancer mechanisms of PPAR ligands might lie in their capacity to disrupt this particular symbiosis

Targeting tumor stroma by drugs not primarily referred to as anticancer drugs and including COX-2 inhibitors, mTOR antagonists and PPAR*γ* agonists has been previously proposed [44–46]. PPAR*γ* actually displays antiangiogenic properties which have been proposed to disrupt the symbiosis between the tumor and the host cells in the tumor bed [46] and which impact negatively several proangiogenic factors among which VEGF, and *β*FGF [46, 47]. Phosphorylation of Erk5 by its activating kinase MEK5 has been recently shown to activate PPAR*γ* and hence to trigger antiangiogenetic signaling [48]. On the other hand, PPAR*γ* has been shown to interfere negatively with VEGF signaling, lowering VEGF-dependent PKC*α* activation of CREB and expression of COX-2 [49].

New advance in the symbiosis existing between the tumor and its stroma has been recently obtained from highlighting the key role played by autophagy in tumor-stroma metabolic crosstalks [50, 51] and on this basis from encouraging anticancer activity of autophagy modulators [52–58]. These modulators not only include mTOR antagonists but also a variety of other pharmacological approaches based on either stimulation or inhibition of autophagy [54–56, 58]. It is currently thought that dysregulated (up- or downregulated) autophagy is detrimental for the permissive complicity existing between tumor and its stroma, the role of autophagy in stromal-epithelial metabolic coupling in cancer becoming better understood [50]. The new anticancer strategies addressing tumoral stroma and metabolic coupling interfere with and take place in the Warburg's phenotype [51]. Like autophagy modulators, modulation of PPAR*γ* antiinflammatory and antiangiogenic signaling might belong to tumoral stroma-directed anticancer therapy.

Although currently not included in clinical trials for anticancer therapy, the use of PPAR*α* and agonists might also be worthy in targeting tumor stroma through antiangiogenic properties. Antiangiogenic properties of the PPAR*α* agonist fenofibrate were initially described with a special emphasis on their protective role against atheromatosis development [59]. More recently, this fibrate has been shown to block tumoral growth through inhibition of angiogenesis [60] in a work coming in the wake of the observation that PPAR*α* deficiency in the host stroma inflammatory cells also suppressed tumoral cell growth [61]. By analogy with autophagy, forced up- or downregulations of PPAR*α* function by genetic manipulation or drug intervention would be detrimental for tumor-host stroma symbiosis through here dysregulation of angiogenesis pathways. Interestingly, another fibrate, clofibric acid for which clinical use was historically suspended because of unexplained deaths and of promoting effects on carcinogenesis, was also recently shown to depress tumoral growth through inhibition of angiogenesis [62]. Recent evidence for the involvements of PPAR*α* signaling in anti-angiogenesis properties resulting from inhibition of either vascular NADP oxidase 1 [63] or epoxidase which takes place in arachidonate metabolism [64] strengthens further the interest of PPAR*α* ligands in disrupting tumor-host stroma symbiosis through antiangiogenic (and anti-inflammatory) mechanisms.

PPAR*β*/*δ* physiologically increases vascular supply through VEGF and other signaling of targeted cells and tissues including skeletal muscle and heart [65–68]. The proangiogenic properties of PPAR*β*/*δ* make this nuclear receptor a new pharmacological target to face ischemic events which may affect heart [69, 70] and brain [71] but also other tissues such as kidney [72]. Regarding tumoral growth, physiological proangiogenic properties of PPAR*β*/*δ* might be regarded as undesired properties, potentially strengthening metabolic coupling between tumor and its stroma, and several experimental and clinical reports have incriminated a positive link between PPAR*β*/*δ* and tumoral cell growth [73–76]. However, recent evidence highlights that PPAR*β*/*δ* and its ligands may also convey, on the opposite, antiangiogenic properties [77, 78].

## 7. PPAR Ligand-Mediated Metabolic Changes Influencing Cancer Development

The anticancer properties of several PPAR ligands and effects have been described in experimental models and more recently in human patients [5, 79, 80]. Several putative underlying mechanisms have been reported, addressing essentially cancer cell signalling, cycle, fate, and life/survival balance determinants [1, 3, 79, 80]. To a lesser extent, the abilities of PPARs to disrupt the metabolic events that typically take place in cancer cells and the tightly related changes induced by biased signaling pathways were also emphasized [1–7, 80–83]. In this respect, the general anticancer effects exerted by PPAR*α* on cellular metabolism and inflammation were recently reviewed by Grabacka and Reiss [84]. The anticancer effects of interest highlighted by these authors included PPAR cooperation with AMP-dependent protein kinase, repression of AKT-driven oncogenicity, inhibition of cell proliferation, transrepression of inflammatory transcription factors, and PPAR transactivation properties leading to overexpressed UCPs and “forced” metabolic catastrophe [84]. In the present review, special attention is paid to the metabolic changes that directly result from PPAR activation and ligands and that interfere with Warburg's effect. The aspects presented here are far from exhaustive and those developed in this section and in a larger manner in this review may be complemented by aspects developed in the other articles mentioned throughout this review.

General therapeutic interventions that interfere with tumoral metabolism have been described above and illustrated in Figure 3(c). In this figure, the metabolic basis for targeting mitochondrial and not nuclear DNA is emphasized. As also mentioned above, ^18^F-deoxyglucose used in PET scan imagery can enter the cell where it is phosphorylated into its phosphate derivative without being further processed. 2-Deoxy-D-glucose may mimic the dissociative effect that glucose-6-phosphate exerts on the hexokinase II/VDAC complex. This type of glucose analogues is currently subject to pharmacological development, and targeting the dissociation of the hexokinase-VDAC complex is an emerging anticancer therapy [85].

### 7.1. PPAR*α* Metabolic Changes and Warburg's Effect

**Figure 7:**
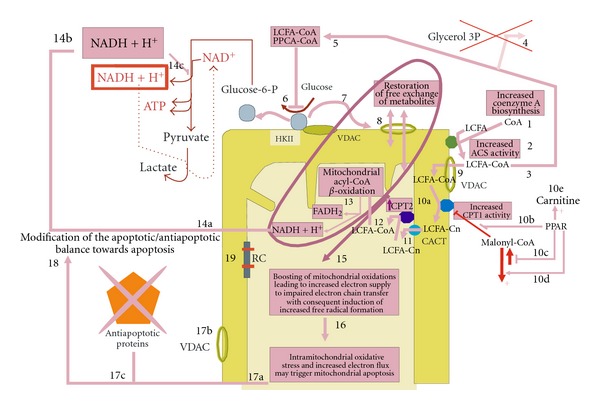
Potential anticancer value of metabolic changes interfering with Warburg's effect and mediated essentially by PPAR*α* (the metabolic events numbered in the figure are explained in the text).

Though PPAR*α* agonists were initially described to convey antiapoptotic properties [86–90], PPAR*α*-driven proapoptotic mechanisms have been also described [91–93]. Mechanisms by which the metabolic action of PPAR*α* might interfere with the Warburg's effect and induce a pro-apoptotic issue are illustrated in Figure 7. This figure provides the reader with a sketch in which direct metabolic effects presented thereafter about PPAR*α* and ligands move to lead to anticancer activity.

Figure 7 is an attempt to group under the same scenario several of PPAR and PPAR ligand-mediated direct effects on intermediary metabolism, notably those induced by PPAR*α*. The scenario is based on integration of several PPAR-driven metabolic features depicted in this figure (for the underlying cell signaling pathways, see the literature cited in this review). 

Referring thereafter directly to the steps numbered in Figure 7, these events include (1) the enhanced cell synthesis and levels of coenzyme A (PPAR*α*) along with (2) increased mitochondrial acyl-CoA synthetase (ACS) activities (PPAR*α*) favours (3) the formation of long-chain fatty acyl-CoAs for which the accumulation(4) is strengthened by deficiency of their removal *via* glycerol esterification (deficient glycerol 3P dehydrogenase in cancer cells), (5) and is known to inhibit hexokinase forms (metabolic regulation). (6) The inhibition of hexokinase II leads to (7) its detachment from VDAC which consequently (8) becomes “re-opened” restoring transfer of a lot many of small water metabolites (particularly those negatively charged and with a molecular weight inferior to 1.5 kDa) from a part to another of mitochondrial outer membrane. (9) More particularly, long-chain acyl-CoA esters (which are relatively soluble in water in comparison to unesterified fatty acids, have a molecular weight lesser than 1.5 kDa, and are negatively charged because of the content of CoA in phosphate functions) may *via* opened VDAC enter mitochondrial intermembranar space. (10) In this space fatty acyl-CoA are converted to their acylcarnitine esters through action of carnitine palmitoyltransferase type I (CPT1) for which catalytic activity facing mitochondrial intermembrane space is enhanced as a result of (10a), its upregulation by PPAR; (10b), reduced malonyl-CoA levels (resulting from reduced formation (10c) and increased decarboxylation (10d)), and increased synthesis and import of carnitine (10e). Reduced malonyl-CoA levels result in an alleviation of inhibitory action on CPT1 which normally occurs at the site located at the cytosolic aspect of acyltransferase (this explains that in untreated cancer cells, high levels of malonyl-CoA may inhibit CPT1 because this effect does not require that the CoA ester transit by VDAC. In contrast, *β*-oxidation of long-chain acyl-CoA requires entry of CoA esters first in intermembrane space *via* transit by VDAC). (11) Fatty acyl-carnitines enter mitochondrial matrix *via* carnitine acylcarnitine translocase (CACT) located in mitochondrial inner membrane. (12) They are then converted back to CoA esters by carnitine palmitoyltransferase 2 (also upregulated by PPAR ligands) to undergo chain shortening by (13) Mitochondrial *β*-oxidation enzymes which are (acyl-CoA dehydrogenases, notably) upregulated by PPARs. The intramitochondrial pathway generates cofactors in their reduced forms (NADH and FADH_2_), and doing so induces an increased electron flux towards impaired respiratory chain. (14) The NADH (and protons) which accumulate locally as a result of their enhanced production (mitochondrial *β*-oxidation) and impaired management (deficient respiration): (14a) may be transferred via oxalate-malate shuttle system to cytosol and (14b) subsequent additional rise in cytoplasmic compartment (14c) may hamper seriously glycolysis to proceed at the level of glyceraldehyde dehydrogenase (NADH-forming) step. (15) The intramitochondrial *β*-oxidation-driven rise in producing reduced cofactors along with impaired respiratory electron chain transfer results in generation of free radicals and other oxidant species. This result is further strengthened by the sudden general boosting of mitochondrial metabolism which may emerge from unlocking VDAC and hence supply of mitochondrial oxidations by massive amounts of small metabolites (see events contained in the oval associated with an arrow pointing close to number 15). (16) This intramitochondrial boosting of metabolism, oxidative stress, and electron flux create conditions favourable to trigger apoptosis. (17) These intramitochondrial (17a) along with other pro-apoptotic events (including (17b), VDAC in its free form and then its potential availability for permeability transition pore, and (17c), downregulation of anti-apoptotic factors by PPAR*γ* ligands) (18) may act on proapototic/antiapoptotic balance towards apoptosis. (19) Impaired respiratory chain (RC) favours intramitochondrial free radical formation and notably direct transfer of electrons to molecular oxygen to form superoxide radical anion *via* radical intermediates of electron transfer chain.

Regarding the dissociation of the hexokinase-VDAC complex mentioned above to be an emerging anticancer issue, it is well known that the diverse hexokinase forms can be inhibited by the CoA esters of long-chain fatty acids [94, 95]. Two metabolic effects induced by PPARs (PPAR*α*) favor the synthesis of long-chain fatty acyl-CoAs: upregulation of mitochondrial fatty acyl-CoA synthetase [96–98] and enhanced cell biogenesis and levels of its cofactor coenzyme A [99]. The latter PPAR effect is consecutive to the upregulation of pantothenate kinase 1 which catalyzes the rate-limiting step of coenzyme A synthesis. These two metabolic features explain how synthesis of long-chain acyl-CoAs is increased following activation of PPAR*α* although one should not overlook the possibility that PPAR*β*/*δ* isoform may upregulate another cellular acyl-CoA-synthetase which is involved in lipid biosynthesis [100]. The PPAR-driven increase in cell fatty acyl-CoA levels is further strengthened secondarily to upregulation by PPAR*α* [101] and PPAR*γ* [102] of acyl-CoA binding protein which is known to stimulate acyl-CoA synthetase activity by removing (binding) the enzyme product [103, 104]. This increase in long-chain acyl-CoAs is also favored by the relative lack of glycerol 3-P which normally branches off glycolysis *via* glycerol 3P dehydrogenase, the latter being deficient in cancer cells [35] as mentioned above. The impairment of this glycerol esterification pathway shifts the acyl-CoA esterification/oxidation balance towards oxidation.

Long-chain fatty acyl-CoAs produced by mitochondria are formed in the vicinity of the VDAC-HKII complex since in cancer cells mitochondrial acyl-CoA synthetase and this complex are both located in the mitochondrial outer membrane. As a result of their inhibitory properties towards hexokinase forms, a local rise in the concentration of long-chain acyl-CoAs should logically inhibit HKII. This effect might be also induced by CoA esters of the pharmacological carboxylic ligands of PPARs. Previously described CoA esters of PPAR ligands include fenofibroyl-CoA, nafenopin-CoA, ciprofibroyl-CoA, and bezafibryl-CoA [105–107].

Importantly, inhibition of HKII activity is classically known to induce its dissociation from VDAC. VDAC, which is closed during its interaction with HKII, then reopens when hexokinase detaches. Since VDAC is involved in the transport of long-chain acyl-CoAs through the mitochondrial outer membrane [34], the opening of this channel unlocks the access of long-chain acyl-CoA to the mitochondrial intermembrane space. In this space, long-chain acyl-CoAs are converted to their carnitine esters by mitochondrial outer membrane carnitine palmitoyltransferase 1 which has its catalytic center facing intermembrane space in contrast to its malonyl-CoA-binding site which is located at the cytosolic aspect of the mitochondrial outer membrane [108, 109]. This acyltransferase may be stimulated following PPAR activation for several reasons, including upregulation by PPAR*α* and *γ* agonists [110–112] and the PPAR*α*-driven release of the inhibition exerted by malonyl-CoA. The release of the inhibitory action of malonyl-CoA on carnitine palmitoyltransferase 1 involves the contribution of PPAR*α* toward the downregulation of acetyl-CoA carboxylase (malonyl-CoA forming enzyme) [113] and the upregulation of malonyl-CoA decarboxylase (malonyl-CoA catabolizing enzyme) [114]. Other PPAR*α*-driven metabolic changes which contribute to stimulate carnitine-dependent entry of fatty acids in mitochondria are the upregulation of both carnitine synthesis and its transport across the cell membrane [115, 116].

Long-chain acyl-carnitines are transferred from the mitochondrial intermembrane space to the mitochondrial matrix *via* the action of carnitine acylcarnitine translocase which belongs to the mitochondrial inner membrane. Within mitochondria, fatty acylcarnitines are converted back to their CoA esters by carnitine palmitoyltransferase 2 which is also the product of an upregulated target gene of PPAR*α* [117]. Fatty acyl-CoAs produced by this acyltransferase are oxidized on the inner side of the mitochondrial inner membrane (very long-chain acyl-CoA dehydrogenase and trifunctional protein) and finally in the matrix. Intramitochondrial *β*-oxidation enzymes, very long-chain [118] and medium-chain acyl-CoA dehydrogenases [119] are themselves also upregulated by PPAR*α* ligands.

In cancer cells with relatively inactive mitochondria, stimulation of both the supply of acyl-CoA and increased *β*-oxidation rates boosts the metabolism in these organelles. The huge increase in the electron flux towards the respiratory chain, often impaired in cancer cells, cannot be handled adequately by tumoral mitochondria resulting in a rise in free radicals and other oxidant species. This oxidative stress may then trigger mitochondria-induced apoptosis.

The anticancer potentialities of these PPAR effects concern cancer cells with so-called lazy mitochondria and may not address all cancer cell lines. Indeed, some cancer cell lines exhibit enhanced mitochondrial *β*-oxidation rates with increased UCP-driven uncoupled accelerated electron transfer rates. In these cases, it has been proposed to be opportune, on the opposite, to act therapeutically by inhibiting [120] rather than stimulating mitochondrial fatty acid oxidation. In cancer cells, the pro-apoptotic basis for “forcing” mitochondrial fatty acid oxidation to proceed at substantial rates might be relatively close to the anticancer mechanisms underlying the effects of mitochondrial membrane permeating compounds such as dichloracetate and pyruvate methyl ester (see above in the text, Figure 3(c)). These two mitocans, by restoring substantial intramitochondrial pyruvate oxidation, are thought to boost the electron flux towards the respiratory chain and hence, in these circumstances, induce an oxidative stress which triggers mitochondrial apoptosis. In this perspective, mitochondrial *β*-oxidation-inducing PPAR ligands could be also classified as mitocans. Importantly, increased plasma ketone body levels may result from PPAR ligand-driven stimulation of mitochondrial *β*-oxidation in ketone body-producing organs. Increased exposure of cancer cells to circulating or local ketones might also boost the electron flux towards mitochondrial respiration at the level of complex II (ketolysis involves succinyl-CoA; acetoacetate CoA transferase and produces succinate). Whether in cancer cell mitochondria such a load on the ketolytic route might trigger apoptosis remains, however, to be elucidated.

### 7.2. PPAR*γ* Metabolic Changes and Warburg's Effect

The pro-apoptotic properties of PPAR*γ* and its ligands may also favor the commitment of cancer cells towards apoptosis. The ligands may act *via* mechanisms either dependent or independent of PPAR. These mechanisms directly address key signaling players of pro-apoptotic routes and were recently reviewed [83].

Regarding mitochondria and apoptosis, PPAR*γ* agonists were also recently described to probably target, *via* PPAR-independent mechanisms, adenylic nucleotide transporters (ANT) [121]. The mitochondrial permeability transition pore complex also recruits VDAC and seems to require the channel to be available in a form dissociated from HKII.

An interesting point is the increase in fatty acid synthesis which in many cancer cell lines is accompanied by increased fatty acid synthase (FASN) capacity and contributes to tumoral cell development [122–125]. Increased fatty acid content represents a reservoir of precursors for signaling molecules that may promote cancer development. As developed elsewhere [126], free fatty acids including those generated by FASN may be toxic for cells. To overcome fatty acid-driven lipotoxicity, cancer cells overexpress enzymes involved in triacylglycerol synthesis, a pathway which removes cellular fatty acids and stores them in a mobilizable and less toxic form (see [126]). In this respect, it has been suggested that a “lipogenetic benefit” results from an unexpected crosstalk between the tyrosine kinase HER2 (human epidermal growth factor receptor) and FASN [127–133]. In cancer cells, this cross-talk is the basis for alleviating fatty acid-driven toxicity by coupling triacylglycerol synthesis to free-fatty acid formation [130, 134, 135], actually increasing aerobic glycolysis or Warburg's effect by pull effect (the pull effect refers to the ability of a metabolic step or pathway to stimulate the step or pathway that precedes it, the principle being similar to the favored displacement of the equilibrium of a chemical reaction towards the product when it is removed, for instance, by evaporation or chelation, from the reaction medium). PPAR*γ*- and PPAR*γ*-binding proteins are in these conditions upregulated by overexpressed HER2 and activate the lipogenetic triacylglycerol synthesis pathway [130, 134, 135]. Very importantly, this collaboration between HER2 and FASN also takes place out of a pathological context and is of physiological relevance in adipogenesis, that is, proliferation and differentiation of adipocyte cell precursors [130, 136].

### 7.3. PPAR*β*/*δ* Metabolic Changes and Warburg's Effect

Like other PPAR isoforms, PPAR*β*/*δ* exerts antiinflammatory and metabolic properties. The clinical use of PPAR ligands only currently emerges, and this shed lights on the physiological activity of this nuclear receptor. Part of the effects of PPAR*β*/*δ* overlaps those exhibited by either PPAR*α* or PPAR*γ* and includes adipocyte differentiation and improved insulin resistance, stimulated fatty acid oxidation in target tissues (heart and skeletal muscle) [67]. For instance, specific PPAR*β*/*δ* agonists may increase HDL cholesterol or HDL/LDL cholesterol ratio, and may decrease excess circulating triglycerides and insulin levels, and, like agonistic PPAR*α* and PPAR*γ* ligands, can counteract some of the aspects of the metabolic syndrome [67, 137–140]. The events depicted in Figure 7 for PPAR*α* might also partially apply to PPAR*β*/*δ* inasmuch its activation may increase mitochondrial fatty acid oxidation in tissues through changes paralleling somewhat those exerted by PPAR*α* and including upregulations of carnitine palmitoyltransferase type I, carnitine acylcarnitine translocase, and long-chain acyl-CoA dehydrogenase [141, 142]. In this respect, PPAR*β*/*δ* has been shown to enable a metabolic shift from glucose to fatty acid utilization [143]. In this respect also, PPAR*β*/*δ* has been described to enable metabolic compensation in deficient PPAR*α* conditions [144].

Besides PPAR*α*-like effects, PPAR*β*/*δ* also displays PPAR*γ*-like effects in targeting the white adipocytes, favoring cell differentiation, fatty acid oxidative capacity, and insulin sensitivity of adipocytes [67].

### 7.4. PPAR-Dependent and Independent Metabolic Changes Induced by Ligands

An essential point determining the exact pathophysiological roles of PPARs and hence their therapeutic potentialities is to distinguish receptor from “extra-PPAR receptor” effects of PPAR ligands. This point has been reviewed by Scatena et al. [145]. These authors highlighted the inhibition of the respiratory chain, notably complex I, as extra-PPAR activities of ligands thus underlining a weak inhibitory effect for PPAR*α* ligand fibrates associated with both glucose and fatty acid oxidations and low differentiating properties. In contrast, PPAR*γ* ligand thiazolidinediones which mediate stronger inhibition of complex I associated with essential glucose utilization and strong cell differentiating activity. The authors also recall that PPAR ligands were initially described as nongenotoxic carcinogens, at least in rodents. Despite such effects, these ligands appear sometimes but not always to be able to influence cancer development in a way favorable for patients.

### 7.5. PPAR, Metabolic Syndrome and Cancer

**Figure 8:**
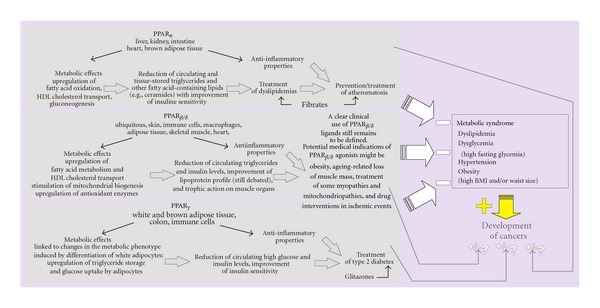
PPARs, metabolic syndrome and cancer. This figure illustrates that (i) PPAR activation may counteract the development of metabolic syndrome, (ii) metabolic syndrome increases the risk for cancer, and (iii) PPARs and their ligands exhibit pro-and antitumoral properties towards cancer progression. Metabolic syndrome is considered as a procancer target antagonised by PPARs, a feature (not further discussed in the text) giving support to the view of a possible preventive anticancer action of PPAR ligands which may be supplied by the diets (polyunsaturated fatty acids, for instance). The figure also compares the links leading to the current medical and pharmacological applications of PPAR ligands.

As illustrated in Figure 8, the tissue distribution and physiological functions of the various PPARs put these nuclear receptors in a privileged position to counteract, through partially overlapping mechanisms, various aspects of the metabolic syndrome. Moreover, cumulated actions of these receptors, as could be achieved by PPAR panagonists, might theoretically jugulate all of the aspects of the metabolic syndrome [146]. On the other hand, metabolic syndrome (defined as a combination of several of the four following traits dyslipidemia, high fasting glycemia, hypertension, and obesity) is currently perceived as a condition which strongly promotes cancer progression [147]. Therefore, the activation of PPARs by preventing or treating the metabolic syndrome removes one important condition promoting tumoral growth though other anticancer mechanisms may be shared by PPARs. As indicated throughout this manuscript, PPAR activation may also trigger signaling that, in contrast, favors tumoral growth. So, the general problem inherent to PPARs and use of PPAR ligands in cancer therapy lies in the fact that pleiotropic effects of these nuclear receptors encompass both cancer brake and accelerator mechanisms. The fact that distinct signaling may be involved in pro- and anticancer properties of PPAR offers the hope to use successfully PPAR ligands in cancer therapy in combination with other active anticancer drugs that overcome the PPAR pro-oncogenic effects. This issue is, however, complicated by the fact that the biological activity of PPAR may be modulated in a cell-or-tissue specific manner and for a same PPAR by the ligand, a phenomenon referred in the literature to SPPARM (selective PPAR modulation) [146], suggesting that PPAR effect in cancers might be cancer dependent. Despite some limits in a wide use of PPAR ligands to treat cancers, clinical trials have been already initiated in this field in recent years. A nonexhaustive presentation of these trials is given in the next section.

## 8. Clinical Trials with Anticancer PPAR Ligands

Clinical trials for evaluating anticancer therapies based on targeting PPAR signaling have been developed against different cancers. For this purpose, drugs that mostly act on the *γ* isoform of PPARs have been used, not necessarily elucidating whether the drugs work via PPAR-dependent mechanisms or *via* PPAR-independent mechanisms due to their chemical structure. The main results obtained to date are given in Table 1. This table is commented below, and the studies described in this table may be completed by general reviews that further stress the therapeutic potential of thiazolidinediones as anticancer drugs, the antineoplastic effects of PPAR*γ* and its role as an antioncogene, the synergetic effects of retinoids in cancer therapy and finally an explanation for the mechanisms underlying the modulation of cancer cell phases [171–181].

In liposarcoma, two phase II trials with PPAR*γ* agonists (either troglitazone or rosiglitazone) [148, 149] indicated cell differentiation of the solid tumors without, however, correlation between drug-induced PPAR*γ* activity in the tumor and the clinical coutcome.

Prostate cancer has been the subject of several clinical trials, and the tumoral growth of human prostate cancer cell lines has also been studied [150–155]. Thiazolidinediones, PPAR*γ* agonists, troglitazone and rosiglitazone, led to conflictual results with two encouraging trials and one trial showing no advantage over the placebo [154]. The deleterious effect of the PPAR*γ* expression and variants were also studied, indicating no association between the Pro12Ala polymorphism and prostate cancer [151], suggesting therefore that PPAR*γ* does not promote prostate cancer development [154]. A study with the LTB4 receptor antagonist LY29311 also known for its PPAR*γ* activating properties failed to demonstrate any efficacy when used in combination with the anticancer drug gemcitabine in advanced prostatic carcinoma [155].

Colo-rectal cancer was also targeted by a clinical phase II trial using the PPAR*γ* agonist thiazolidinedione troglitazone against chemotherapy resistant metastatic colorectal cancer [156]. No objective or negative tumor response was observed in this and other studies [156–158]. Interestingly, in the scope of the Bezafibrate Infarctus Prevention (BIP) study, some experimental support was obtained to suggest preventive effects for bezafibrate (a PPAR panagonist) against the development of colon cancer [159].

A phase II clinical trial using troglitazone in metastatic breast cancer refractory to chemotherapy or hormonal therapies suggested moderate efficacy of this PPAR*γ* agonist [160]. At the same time, preclinical studies indicated that in breast cancer cell lines PPAR*α* expression is dependent on the estrogen receptor and represents a marker for sensitivity/resistance to histone deacylase inhibitors [161]. When PPAR*γ*1 signalling is increased in breast cancer, it impacts the balance between cell death and cell proliferation in favor of tumoral growth [162].

Studies in leukemia suggested that PPAR*γ* regulates apoptosis at the level of caspase 8, and its coactivator DRIP205 was found to promote cell differentiation *via* PPAR*γ* [164]. Sensitization of TRAIL-resistant cells to TRAIL was also reported for the natural PPAR*γ* agonist 15d-PGJ2, however, independently of PPAR*γ* signaling [163].

In thyroid cancer, the synthetic PPAR*γ* agonist rosiglitazone was shown to enhance the uptake of radioiodine by thyroid tumors in a way apparently independent of PPAR*γ* [165, 166]. Although this effect is not per se an anticancer mechanism, it is of therapeutic interest in potentiating the radioiodine-based chemotherapy of hyperthyroidism, thyroid, and other cancers. Interestingly, this potentiating effect indicates that rosiglitazone administration should be theoretically questioned in a context of defective nuclear power plants or in people living close to these plants.

PPAR*γ* targeting in human glioma and glioblastoma has provided encouraging results in *in vitro* and *in vivo* studies when thiazolidinedione is combined with either a statin or a coxib. Yao and coworkers [167] have shown anticancer activity with a combination of lovastatin and troglitazone in glioblastoma and lung cancer cells. The anticancer properties of the exposure of cells to this dual treatment included the combined enhancement of intracellular levels of P27 [Kip 1] (usually induced by statins) and E2F1 (induced by glitazones) along with changes in the status of CDK2, cyclin A and Rb phosphorylation. A phase II study combining the PPAR*γ* agonist pioglitazone and rofecoxib with low-dose chemotherapy in high-grade gliomas pointed out a moderate benefit and encourages the future use of this cocktail in highly selected patients [168].

Head and neck cancers were also challenged with PPAR*γ* ligands with some benefit as reviewed elsewhere by Schweitzer et al. [169].

In melanoma, tumoral cell growth has been shown to be inhibited by the thiazolidinedione ciglitazone, in a way independent of PPAR*γ* activation [170]. This antitumoral activity of ciglitazone has been further shown to involve downregulation of chemokine CXCL1 and microphthalmia-associated transcription factor, MITF, two proteins overexpressed in human and playing a key role promoting its pathogenesis [182].

## 9. Conclusions and Perspective

This review has attempted to account for the rationale underlying the metabolic functioning of cancer cells in relation with Warburg's effect, and its corollaries in terms of metabolic vulnerability. More precisely, the metabolic behavior of cancer cells with lazy mitochondria was emphasized because it theoretically represents a nearly ideal target for PPAR*α* agonists to reverse the cancer cell-driven metabolic lock of mitochondrial metabolism. Nevertheless, the perception of Warburg's effect currently evolves, and in this context defective mitochondrial oxidative capacity is not longer viewed as a mandatory component, substantial mitochondrial oxidative activities contributing in this context to cover cancer cell energetic needs. Anticancer metabolic and other effects presented throughout the text in relation with the Warburg's effect should be completed in more details with anticancer activities exerted by PPAR ligands in many realms other than metabolism, inflammation, and angiogenesis as for instance cell cycle, cell survival, cell maturation, cell differentiation, tumoral invasion, and apoptosis. In this review, the latter process has been further proposed to be sensitive to unlocking of mitochondrial metabolism and respiration induced by PPAR*α* agonists. Surprisingly, clinical trials with PPAR*α* ligands are still to be initiated, possibly because they were initially described to be nongenotoxic carcinogens that could mediate antiapoptotic properties [86–90]. Since then species differences [183] and PPAR*α*-driven pro-apoptotic mechanisms [91–93] have been documented. In contrast, several trials with PPAR*γ* ligands have been conducted to date. PPAR*γ* and their ligands are shown in the literature to favor apoptosis *via* multiple actions on cell-signaling pathways. At the same time, they may also be able to promote anti-apoptotic pathways. This is now well documented for the PPAR*γ* ligand troglitazone which promotes apoptosis *via* upregulation of the TRAIL death receptor, inhibition of the anti-apoptotic proteins FLIP, and downregulation of survivin [184]. In turn, troglitazone may also activate anti-apoptotic pathways, for instance, *via* enhanced phosphorylation of ERK and subsequently of BAD, resulting in increased availability of the anti-apoptotic proteins Bcl-2 and Bcl-X_L_ to scavenge key pro-apoptotic mediators such as BAX [185]. The fact that in this case pro- and anti-apoptotic actions are mediated by separate signaling pathways offers the perspective of potentiating the former by inhibiting the second, and in practice, by combining troglitazone with an inhibitor of the events leading to BAX sequestration *via* the anti-apoptotic proteins mentioned above. The need for this association of PPAR ligands with other anticancer agents is perhaps one of the most important lessons to be learned from clinical trials based on the use of these ligands to treat cancer.

## Figures and Tables

**Table 1:** Clinical trials and some preclinical studies for evaluation of PPARs and their ligands in cancer development and anticancer therapy.

References	Nuclear receptor	Treatment	Clinical phase	Number of patients	Human tumors or cell types	Main conclusions of the authors
Liposarcoma						
Demetri et al., 1999 [148]	PPAR*γ*	Troglitazone (per os, 1 × 800 mg/d, 6 weeks)	Phase II	3	High-grade liposarcomas	Induction of cell differentiation in a human solid tumors
Debrock et al., 2003 [149]	PPAR*γ*	rosiglitazone (4 mg/d for 1 year)	Phase II	12	Liposarcoma	Increased PPAR*γ* activity: no correlation with clinical outcome

Prostate cancer						
Mueller et al., 2000 [150]	PPAR*γ*	Troglitazone (per os, 2 × 400 mg/d, 12 weeks)	Phase II	41	Human prostate cancer	Prolonged stabilization of PSA with PSA close to 0 in 1 patient
Paltoo et al., 2003 [151]	PPAR*γ* Pro12Ala	Impact of a gene polymorphism on prostate cancer development		193 (versus 188 controls)	Prostate cancer	No association of prostate cancer and Pro12Ala polymorphism
Xu et al., 2003 [152]	PPAR*γ*	Tosiglitazone	Preclinical		Primary culture of human prostatic cancer cells	Prodifferentiating properties of thiazolidinediones
Dawson and Slovin, 2003 [153]	Vit D PPAR*γ*	Review			Prostate cancers	
Smith et al., 2004 [154]	PPAR*γ*	Rosiglitazone 2 × 4 mg/d 338 d (versus placebo 315 d)	Phase II	105	Prostate carcinoma without recent hormone therapy and with a rise in PSA after radical prostatectomy and/or radiation without metastasis	No advantage over placebo efficacy and PPAR*γ* do not contribute to prostate cancer development
Saif et al., 2009 [155]	PPAR*γ* agonist and LTB4 receptor antagonist	LY29311 (in combination with gemcitabine)	Phase II	67 (combined therapy) versus 66 (gemcitabine alone)	Advanced pancreatic carcinoma	No benefit obtained by adding LY293111 to gemcitabine

Colo-rectal cancer						
Kulke et al., 2002 [156]	PPAR*γ*	Troglitazone per os	Phase II	25	Chemoresistant colorectal metastatic cancer	No objective tumor response
Choi et al., 2008 [157]	PPAR*γ*	15d-PGJ2 pioglitazone	Preclinical		APC-mutated HT-29 human colon cancer cells	PPAR*γ* ligand promotes growth of APC-muted HT-29 colon cancer cells
Dai and Wang, 2010 [158]	PPAR*γ*	Review			Colorectal cancer	Mechanisms by which PPAR*γ* impacts carcinogenesis in colorectal cancer
Tenenbaum et al., 2008 [159]	All PPARs	Panagonist bezafibrate retard 400 mg/d	Bezafibrate Infarctus Prevention (BIP) study	3011 with coronary artery disease and no cancer	1506 given bezafibrate 1505 given placebo	Experimental support for preventive effects of bezafibrate towards colon cancer
Breast cancer						
Burstein et al., 2003 [160]	PPAR*γ*	Troglitazone 800 mg/d for 6 months	Phase II	22	Breast cancer refractory to one chemotherapy or two hormonal therapies	Little apparent clinical effect in patients with treatment refractory metastatic breast cancer
Faddy et al., 2006 [161]			Preclinical			(1) ER dependence of PPAR*α* (2) PPAR*α* levels = marker of breast cancer cell resistance to histone deacylase inhibitors
Zaytseva et al., 2008 [162]	PPAR*γ*1	RNAi	Preclinical		MCF-7 breast cancer cells	PPAR*γ*1 signaling impacts balance between proliferation and apoptosis towards proliferation in breast cancer

Leukemia						
Hasegawa et al., 2007 [163]	PPAR*γ*	Fuligocondis B *via* increase in PGJ2	Preclinical		Leukemia cells	15d-PGJ2 sensitizes TRAIL-resistant cells to TRAIL independent on PPAR*γ*
Tsao et al., 2010 [164]	PPAR*γ*	2-cyano-3,12-dioxooleana-1,9-dien-28-oic acid (CDDO) 15d-PGJ2	Preclinical Phase I	9	Acute myelogenous leukemia cell lines from 9 patients	PPAR*γ* regulates apoptosis *via* activation of caspase 8 and co-activator DRIP205 promotes cell differentiation by PPAR*γ*

Thyroid cancer						
Kebebew et al., 2006 [165]	PPAR*γ*	Rosiglitazone 4 mg/d 1 week + 8 mg/d 7 weeks	Phase II	10 patients	Differentiated thyroid cancer	Rosiglitazone may induce radioiodine uptake in some patients possibly *via* PPAR*γ*-independent pathways
Tepmongkol et al., 2008 [166]	PPAR*γ*	Rosiglitazone 8 mg/d for 6 weeks	Phase II	23	Thyroid carcinoma	Increase of radioiodine uptake in thyroid tumors possibly *via* PPAR*γ*-independent mechanisms

Glioma and glioblastoma, lung cancer						
Yao et al., 2006 [167]	HMG-CoA reductase PPAR*γ*	Lovastatin + troglitazone	Preclinical		Glioblastoma and lung cancer cell lines	Induction of P27 [Kip 1] (statines), E2F-1 (glitazone), + CDK2, cyclin A. Rb phosphorylation status
Hau et al., 2008 [168]	COX-2 inhibitors PPAR*γ*	Rofecoxib pioglitazole in association with low-dose chemotherapy	Phase II	44	High-grade gliomas (glioblastomas or anaplastic glioma)	Moderate activity encouraging future utilization in highly selected patients

Head and neck cancer						
Schweitzer et al., 2010 [169]	PPAR*γ*	Review			Head and neck cancers	Therapeutic use of PPAR*γ* ligands in head and neck cancer

Melanoma						
Botton et al., 2009 [170]	PPAR*γ*	Ciglitazone versus other thiazolidinediones	Preclinical		Melanoma cell lines	Effects on cycle arrest, p21, cyclin D1, pRB hypophosphorylation are better than with other thiazolidinediones
